# The common ancestry of life

**DOI:** 10.1186/1745-6150-5-64

**Published:** 2010-11-18

**Authors:** Eugene V Koonin, Yuri I Wolf

**Affiliations:** 1 National Center for Biotechnology Information, National Library of Medicine, National Institutes of Health, Bethesda, MD 20894, USA

## Abstract

**Background:**

It is common belief that all cellular life forms on earth have a common origin. This view is supported by the universality of the genetic code and the universal conservation of multiple genes, particularly those that encode key components of the translation system. A remarkable recent study claims to provide a formal, homology independent test of the Universal Common Ancestry hypothesis by comparing the ability of a common-ancestry model and a multiple-ancestry model to predict sequences of universally conserved proteins.

**Results:**

We devised a computational experiment on a concatenated alignment of universally conserved proteins which shows that the purported demonstration of the universal common ancestry is a trivial consequence of significant sequence similarity between the analyzed proteins. The nature and origin of this similarity are irrelevant for the prediction of "common ancestry" of by the model-comparison approach. Thus, homology (common origin) of the compared proteins remains an inference from sequence similarity rather than an independent property demonstrated by the likelihood analysis.

**Conclusion:**

A formal demonstration of the Universal Common Ancestry hypothesis has not been achieved and is unlikely to be feasible in principle. Nevertheless, the evidence in support of this hypothesis provided by comparative genomics is overwhelming.

**Reviewers:**

this article was reviewed by William Martin, Ivan Iossifov (nominated by Andrey Rzhetsky) and Arcady Mushegian. For the complete reviews, see the Reviewers' Report section.

## Background

In the *Origin of Species*, Charles Darwin famously proposed what we may now call the Universal Common Ancestry (UCA) hypothesis: "I should infer from analogy that probably all the organic beings which have ever lived on this earth have descended from some one primordial form, into which life was first breathed." [1]. For a century after the publication of Darwin's bold proposition, before the advent of molecular biology, the UCA hypothesis remained an untested and hardly testable speculation. However, first the universality of the genetic code and later the demonstration of the (near) universal conservation of approximately 100 RNA and protein-coding genes among cellular life forms provided ample evidence in support of the UCA [2,3]. Although generally considered compelling, this evidence fell short of a rigorous, formal test of the UCA hypothesis.

In a recent, remarkable Letter to Nature, Theobald applied an information-theoretical approach to offer just that: a formal, homology-independent test for the hypothesis of the common ancestry of the extant cellular life forms [4], a claim that is further reaffirmed in the accompanying News and Views article by Steel and Penny [5]. Following the general information theoretical framework for statistical tests of common ancestry laid out previously by Sober and Steel [6], Theobald reports a likelihood ratio test of the common ancestry hypothesis for genes represented by orthologs in the three domains of life. According to Theobald, "...when comparing a common-ancestry model to a multiple-ancestry model, the large test scores are a direct measure of the increase in our ability to accurately predict the sequence of a genealogically related protein relative to an unrelated protein." [4]. It is interesting to note that this "formal demonstration of the common ancestry of life" seems to quickly gain quite some following. Thus, the Wikipedia article on the Last Universal Ancestor quotes Theobald's study as the principal argument in support of the UCA [7].

We maintain, however, that the purported formal demonstration of the Universal Common Ancestry of all known cellular life forms is illusory. Indeed, in the quoted key sentence, the claim that the sequence of one of the universal proteins (e.g., a bacterial version) predicts another (e.g., the corresponding archaeal version) is simply a restatement of the fact that these proteins display a highly statistically significant sequence similarity.

## Results

**Figure 1:**
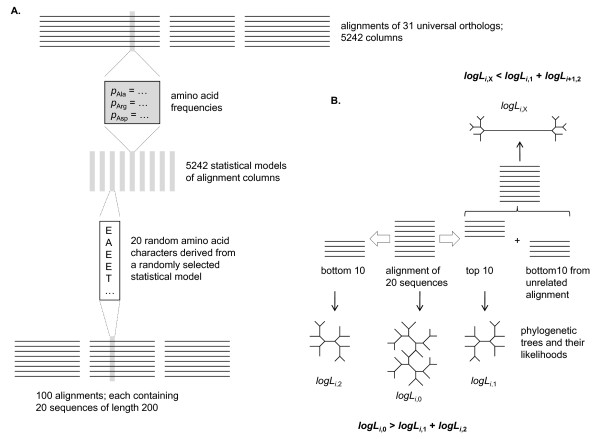
**The computational experiment on the estimation the likelihoods of alignments of similar but evolutionarily unrelated sequences**. (A) Generation of random alignments from statistical models of real alignment columns (alignment columns containing >50% of gap characters were removed). (B) Construction of phylogenetic trees from random alignment halves and likelihood analysis.

To formally demonstrate the independence of Theobald's test on the common ancestry of the compared sequences, we designed and performed the following computational experiment (Figure 1). We derived a statistical model (frequencies of amino acids) for each of the 5242 columns in the alignment of universally conserved proteins (primarily those involved in translation) used by Ciccarelli et al. to reconstruct a "Tree of Life" [8,9]. Then we constructed 100 alignments each of which contained 20 sequences of length 200. Each column of these alignments was generated by randomly selecting a statistical model (from the set of 5242 models) and then emitting 20 random amino acid characters with probabilities derived from the chosen model. In each of the 100 generated alignments, all sequences are highly similar to each other because each alignment column is derived from a single statistical model. However, these alignments contain no signal of common ancestry (in more general terms, no evolutionary signal) whatsoever because each position in each sequence is generated independently from other positions.

We then split each alignment into two alignments of 10 sequences each and reconstructed phylogenetic trees for each of the resulting 300 alignments (100 original ones plus two halves for each; Figure 1) using the PhyML program (WAG evolutionary model, alpha parameter of gamma distribution set to 1) [10]. Log likelihood values reported by PhyML were collected for each original alignment and for its two halves. In all 100 cases, the log likelihood of the combined alignment was higher than the sum of log likelihoods of the halves (difference ranged from +432 to +565 log likelihood units with the mean of +506). The difference in log likelihoods accumulated over 100 alignments amounted to the combined alignments being more likely than an independent emergence of the two halves by a factor of ~10^21993^, a value that leaves no doubts about its significance.

In contrast, when unrelated alignments were merged (first half of alignment #1 with the second half of alignment #2 etc), all log likelihoods from combined alignments were substantially lower than the sum of individual log likelihoods (difference ranged from -133 to -45 log likelihood units with the mean of -90). In this case, the lower likelihood of the combined alignment reflects the improbability of a very long tree branch connecting two dissimilar clusters of sequences.

This experiment demonstrates that the phenomenon observed by Theobald [4] is, indeed, entirely a product of "our ability to accurately predict the sequence of a... related protein relative to an unrelated protein" regardless of the actual history of the corresponding sequences. Alignments of statistically similar but phylogenetically unrelated sequences successfully mimic the purported effect of common origin. Thus, the nature and origin of the similarity between the aligned sequences are irrelevant for the prediction of "common ancestry" of proteins under Theobald's approach. Accordingly, common ancestry (or homology, in the modern, post-Darwinian sense) of the compared proteins remains an inference from sequence similarity rather than an independent property demonstrated by the likelihood analysis.

## Discussion

The tests described above show that there is currently no formal demonstration of the universal common ancestry of the extant life forms. The likelihood tests of the kind described by Theobald [4] fail to address the problem because they yield results "in support of common ancestry" for any sufficiently similar sequences. The alternative to UCA is convergent evolution of highly similar sequences of the universal proteins (under the convergence hypothesis, the phrase "universally conserved" becomes an oxymoron). The plausibility of the convergence hypothesis depends on the strength of constraints that affect evolutionary trajectories of isofunctional proteins [11]. Several lines of evidence indicate that convergence is not a viable explanation for the extensive sequence similarity that is observed among universal proteins. First, the available experimental studies, however limited, suggest that, although only a small fraction of the vast sequence space is open for evolution, the available trajectories are nevertheless numerous, so that evolution is far from deterministic [12,13]. Second, the few described cases of actual convergent evolution of similar protein sequences, resulting in independent emergence of the same enzyme specificity, involve only a few key amino acid residues and do not attest to convergent origin of highly similar sequences [14,15]. Third, and perhaps most convincing, for about 10% of the known enzyme specificities, isofunctional proteins without detectable sequence or structure similarity have been detected, an indication that multiple, independent solutions to the same biological function are accessible to evolution [16,17].

We believe that together this evidence makes convergent evolution of the highly similar sequences in over 100 proteins that are confidently traced back to the putative Last Universal Cellular Ancestor (a highly conservative estimate) [2] a virtual impossibility. However, formal demonstration of UCA, independent of the assumption that universally conserved orthologous proteins with highly similar sequences actually originate from common ancestral forms, remains elusive and might not be feasible in principle.

## Competing interests

The authors declare that they have no competing interests.

## Authors' contributions

EVK initiated the study, EVK and YIW designed research, YIW performed research, EVK and YIW wrote and edited the manuscript.

## Reviewers' comments

### Reviewer 1

William Martin, University of Duesseldorf

Here, Koonin and Wolf show that "the purported demonstration (by Theobald [4]) of the universal common ancestry is a trivial consequence of significant sequence similarity between the analyzed proteins". They are absolutely right on this in my view and there is not much more to say about this set of circumstances, really. The issue is a recent paper by Theobald claiming to have found evidence for common ancestry of life based on the analysis of 23 sequences that Jim Brown and colleagues [18] had identified as "universal" among genomes on the basis of database searches and sequence comparisons, but 10 years ago (in 2001). One might addd that in 2000, Hansmann and Martin showed that the same proteins that Theobald investigated are even encoded in the same superoperon in most prokaryotes [19]. One wonders whether Theobald should have perhaps commented on the "razor sharp" intellectual insight of those 2000 and 2001 authors to infer that the proteins that they identified are related via common ancestry (because of obvious sequence similarity), the conclusion in the title of Theobald's paper. Cogniscenti cringed when they saw the Theobald paper, knowing that "it is trivial". It is trivial because the straw man that Theobald attacks in a text largely formulated in convoluted legalese, is that significant sequence similarity might arise by chance as opposed to descent with modification. Ignoring the strength of the universality of the genetic code and the commonality of central intermediary metabolism among cells as evidence, Theobald construed a non-issue that the referees of his paper, whoever they may have been, found convincing and novel (!).

Here, Koonin and Wolf reexamine the issue from an independent standpoint and find that Theobald's result "is simply a restatement of the fact that these proteins display a highly statistically significant sequence similarity". I could not agree more and recommend that this crisp paper go to publication in present form.

### Reviewer 2

Ivan Iossifov, Cold Spring Harbor Laboratory, nominated by Andrey Rzhetsky

In this manuscript Koonin and Wolf critique the model-selection method used by Theobald in a recent Nature publication to formally prove the UCA (Universal Common Ancestor) hypothesis. The main argument is that the method fails to differentiate between the UCA hypothesis and convergent evolution hypothesis. The authors perform a simulation experiment which clearly demonstrates their point--the Theobald's method chooses UCA hypothesis with virtual certainty over data generated by a convergent evolution model. The critique is of only theoretical nature---as the authors themselves state (and provide strong evidence for), the convergent evolution is not a viable alternative to UCA. I find the manuscript to be concise, clear, and well written. I do believe that the theoretical discussion would be of interest to the mathematical biology community.

### Reviewer 3

Arcady Mushegian, Stowers Institute for Medical Research

The question that Koonin and Wolf address in this communication is important in its own right, and also became a hot topic after publication of D. Theobald's *Nature *paper earlier this year. Even a brief communication in Biology Direct should be self-sufficient, so I think that the first thing the authors of the current study should do is to explain what Theobald did in his work, not only what he claimed to have done. Perhaps the proper place to start may be an earlier paper by Sober and Steel [6]. Theobald cites it but does not mention that his own work is, to an extent, the attempt to realize the proposal by Sober and Steel.

Response: *We agree and incorporated this point into the revised version of the article: "Following the general information theoretical framework for statistical tests of common ancestry laid out previously by Sober and Steel *[6], *Theobald reports a likelihood ratio test of the common ancestry hypothesis for genes represented by orthologs in the three domains of life."*

Discussion of all this, by both Theobald and Koonin&Wolf, is a bit confusing, at least to this reviewer, in at least two respects. First, it is unclear what hypothesis is being tested in either of the two papers. Is it a null hypothesis testing sensu Fischer, or testing of two alternate hypotheses sensu Neyman-Pearson, and in either case, what the hypothes(i/e)s is/are?

Response: *A rather subtle issue but we are inclined to interpret these tests within the null hypothesis framework*. *The hypotheses are stated quite explicitly by both Theobald and ourselves: the general null hypothesis of independent ancestry and the specific hypothesis of common ancestry*.

Second, I have a problem with multiple statements in both papers about this or that thing not being dependent on the hypothesis of the common ancestry. It does not require any simulation to point out, e.g., a flaw of this class in Theobald's work, when he says one cannot conclude anything direct about common ancestry from BLAST P-value, and has to infer it somehow. Surely, one must know that the inference in this case is possible because Karlin-Altschul statistics relies on the scoring system (s parameter) that is derived from the large dataset of alignments of bona fide homologous proteins! This and other examples seem to indicate that Theobald's argument may be based on tautology. Can the authors elaborate on whether their simulation is testing the circularity of the argument (and whether it is even able to do so, as the simulation itself is also not completely devoid of the evolutionary signal, having been built by sampling from the models that are derived from alignments of orthologs), or is it doing something else?

Response: *We tend to disagree: the whole point is that the shuffled alignment columns in our test carry a signal of sequence similarity but not an evolutionary signal. Although each column in the shuffled alignment originates from an alignment of homologous sequences, the statistical models do not depend on this fact and do not actually retain the information of the evolutionary relatedness of the respective genes but rather could have been generated a priori. We clarify this in the revision*.

I would also be cautious asserting that there may be no good statistical test for the common ancestry in principle. For example, we may try to use a joint probability of several events occurring independently, to show that such probability is infinitesimally low, even if the probability of each individual event is not particularly low. One such collection of events may be the preponderance of non-omnipresent, but widely distributed in diverse organisms, genes that were not used to build the consensus Tree of Life, but whose own phylogeny is compatible, or at least very close, to such a tree.

Response: *We do not "assert" the infeasibility of this kind of test. However, we currently cannot think of a schema that would allow it, so we cautiously point out that such a test "might not be feasible in principle"*.

Finally, some more mileage can be gotten out of explicitly rejecting the hypothesis of the convergent or parallel origin of similar protein sequences, even if not in the sense of statistical hypothesis testing, but in the more lay sense of stating that all the evidence points in the opposite direction, and none of the examples of long-range sequence convergence withstand the scrutiny. I agree with all arguments that the authors present here, but I wish that they extend their argument and mention and perhaps discuss the essay by R.F. Doolittle [11], where this is laid out in more detail.

Response: *We do not see a need to elaborate much but Doolittle's paper is cited in the revision*.
